# Reference Intervals for Hematology and Clinical Chemistry for the African Elephant (*Loxodonta africana*)

**DOI:** 10.3389/fvets.2021.599387

**Published:** 2021-03-01

**Authors:** Christine Steyrer, Michele Miller, Jennie Hewlett, Peter Buss, Emma H. Hooijberg

**Affiliations:** ^1^Department of Companion Animal Clinical Studies, Faculty of Veterinary Science, Centre for Veterinary Wildlife Studies, University of Pretoria, Pretoria, South Africa; ^2^Division of Molecular Biology and Human Genetics, Department of Science and Innovation-National Research Foundation Centre of Excellence for Biomedical TB Research, Faculty of Medicine and Health Sciences, South African Medical Research Council Centre for Tuberculosis Research, Stellenbosch University, Cape Town, South Africa; ^3^Department of Production Animal Studies, Faculty of Veterinary Science, Centre for Veterinary Wildlife Studies, University of Pretoria, Pretoria, South Africa; ^4^Veterinary Wildlife Services, South African National Parks, Skukuza, South Africa

**Keywords:** African elephant, hematology, reference intervals, VetScan VS2, serum chemistry, *Loxodonta africana*, leukocyte morphology

## Abstract

The African elephant (*Loxodonta africana*) is listed as vulnerable, with wild populations threatened by habitat loss and poaching. Clinical pathology is used to detect and monitor disease and injury, however existing reference interval (RI) studies for this species have been performed with outdated analytical methods, small sample sizes or using only managed animals. The aim of this study was to generate hematology and clinical chemistry RIs, using samples from the free-ranging elephant population in the Kruger National Park, South Africa. Hematology RIs were derived from EDTA whole blood samples automatically analyzed (*n* = 23); manual PCV measured from 48 samples; and differential cell count results (*n* = 51) were included. Clinical chemistry RIs were generated from the results of automated analyzers on stored serum samples (*n* = 50). Reference intervals were generated according to American Society for Veterinary Clinical Pathology guidelines with a strict exclusion of outliers. Hematology RIs were: PCV 34–49%, RBC 2.80–3.96 × 10^12^/L, HGB 116–163 g/L, MCV 112–134 fL, MCH 35.5–45.2 pg, MCHC 314–364 g/L, PLT 182–386 × 10^9^/L, WBC 7.5–15.2 × 10^9^/L, segmented heterophils 1.5–4.0 × 10^9^/L, band heterophils 0.0–0.2 × 10^9^/L, total monocytes 3.6–7.6 × 10^9^/L (means for “regular” were 35.2%, bilobed 8.6%, round 3.9% of total leukocytes), lymphocytes 1.1–5.5 × 10^9^/L, eosinophils 0.0–0.9 × 10^9^/L, basophils 0.0–0.1 × 10^9^/L. Clinical chemistry RIs were: albumin 41–55 g/L, ALP 30–122 U/L, AST 9–34 U/L, calcium 2.56–3.02 mmol/L, CK 85–322 U/L, GGT 7–16 U/L, globulin 30–59 g/L, magnesium 1.15–1.70 mmol/L, phosphorus 1.28–2.31 mmol/L, total protein 77–109 g/L, urea 1.2–4.6 mmol/L. Reference intervals were narrower than those reported in other studies. These RI will be helpful in the future management of injured or diseased elephants in national parks and zoological settings.

## Introduction

The African elephant (*Loxodonta africana*) is a megaherbivore, which had an extensive range across the African continent until the 1930s. Loss of habitat and poaching led to the present classification of this species as Vulnerable by the International Union for Conservation of Nature (IUCN) (1). The total population on the continent is presently estimated to be around 415,000 individuals (2). According to the African Elephant Status report, the minimum estimated total number of individuals within South Africa is 18,841, with the biggest population, of at least 17,086 individuals, living in the Kruger National Park (KNP) (2). The subspecies of this population can further be classified as *Loxodonta africana africana* (South African bush elephant) based on the geographical distribution (3). Although this population is considered stable, poaching is a threat. According to South African National Parks (SANParks), 71 elephants were poached and killed in the country in the year 2018, and 31 elephants in 2019 (4). Elephants (Asian and African) are commonly kept in managed (or in human care) settings such as zoos and circuses, including 78 zoos in North America and 114 in Europe (3). According to the Elephant Database, close to 9,000 individuals are recorded to be in these zoo and sanctuary populations; this number does not distinguish between the Asian and the African elephants (5). Additionally, another 16,000 managed Asian elephants are reported to live on the Asian continent (6).

Clinical pathology reference intervals (RI) are one of the most valuable diagnostic tools in veterinary medicine and are used to help differentiate diseased from healthy individuals (7, 8). They are especially important for free-ranging animals, where other diagnostic modalities such as imaging are usually more limited than in practice settings. Published RI studies for African elephants have been performed using managed animals (9, 10) or with blood samples taken from culled animals (11). The analytical methods used in most studies are outdated or not even described (12). More recent papers concerning hematology and clinical chemistry have investigated panels which contain only a few measurands, for example nutritional evaluations in zoological institutions (13, 14). Newer RI studies concern only the Asian elephant (*Elephas maximus*), mainly in Sri Lanka, Thailand and Myanmar, presumably because of the easier access to this species, as these animals are at least partially under human care (15–17). Some data are also available *via* the Species360 Database [former International Species Information System (ISIS)] (18).

The objective of this study was to establish RI for hematology and selected clinical chemistry measurands for a free-ranging African elephant population. The RI were generated in accordance with the guidelines published by the American Society for Veterinary Clinical Pathology (ASVCP) (7) with minor modifications based on information from more recent modeling studies (19, 20).

## Materials and Methods

Ethical approval specifically for this study was obtained from the University of Pretoria Faculty of Veterinary Science Research Ethics Committee and Animal Ethics Committees (certificate number REC 132-19).

The samples for this study originate from the free-ranging African elephant population from the KNP, South Africa. The animals were immobilized for park management purposes or other unrelated studies. Immobilization was performed by Veterinary Wildlife Service (VWS) veterinarians according to SANParks Standard Operating Procedures (SOP). Elephants were darted from a helicopter using an air-pressurized dart (3 mL Dan Inject plastic dart; DAN INJECT, International S.A., Skukuza 1350, South Africa) propelled by a carbon dioxide powered rifle (DAN INJECT JM-special, Skukuza 1350, South Africa). Immobilization was induced with etorphine (Novartis, Kempton Park 1619, South Africa), azaperone (Janssen Pharmaceutical Ltd., Halfway House 1685, South Africa), and hyaluronidase (Kyron Laboratories, Benrose 2011, South Africa), with dose ranges based on subjective weight and age estimation by the same veterinarian (etorphine 0.003 mg/kg and azaperone 0.01 mg/kg). Once the elephant was recumbent, the ground crew approached and assisted the elephant into lateral recumbency, if required. At the end of the procedure, naltrexone (Kyron Laboratories, Benrose 2011, South Africa) was administered intravenously at 20 times the etorphine dose (mg), and the animal observed until it had fully recovered. Sample collection proceeded according to a standardized protocol as follows: blood was taken *via* a 18G needle and direct vacutainer collection from an auricular vein at first handling after the venipuncture site was swabbed with alcohol (ethanol 70%). Disposable medical gloves were worn during the blood collection. Whole blood was collected in sealed EDTA and serum vacutainers (BD Biosciences, Franklin Lakes, NJ, USA). Serum samples were left to clot for at least 30 min standing upright in a cooler box. Samples were transported cooled, until they were processed in the VWS laboratory within 6 h of collection. EDTA whole blood was analyzed with the scil Vet abc (scil Animal Care Company, Ontario, Canada) or the Horiba ABX Micro VS60 (Horiba ABX SAS, Kyoto, Japan) hematology analyser. Automated hematology analysis was not routinely performed for all animals. Blood smears were made using a standard pushing (wedge) technique (21) and stained with a commercial modified Romanowski stain (Kyro-Quick stain, Kyron Laboratories) for manual differential counts. Microhematocrit tubes for determination of packed cell volume (PCV) were prepared using a microhematocrit centrifuge (Model HKT-400, Gemmy Industrial Corporation, Taipei, Taiwan; 15,000 *g*, 5 min). Serum tubes were centrifuged at 1,300 *g* for 10 min, and serum aliquoted into cryotubes (Greiner Bio-One, Lasec S.A., PTY LTD Cape Town, 7405, South Africa) and frozen at −80°C.

At the time of immobilization, a physical examination was performed. Animals without injuries and free of clinical abnormalities were considered healthy. All data for the animal, including the sex, general condition, age and weight estimation, microchip number and geographical location of the immobilization site, were recorded in Excel spreadsheets. Notes were added for abnormal clinical findings or injuries, if present. Sample selection was made according to this information. All selected samples were collected between October 2014 and August 2019, meaning they were stored no longer than 5 years. This threshold was chosen as no studies could be found on stability beyond this time (22). Samples and data from a total of 79 apparently clinically healthy animals were selected for the reference interval study.

### Hematology

Data from the original hematology analyses were reviewed. Analysis was performed using EDTA whole blood after mixing at room temperature, with a Scil Vet ABC (first 25 results) and a Horiba ABX Micros ESV60 (last 11 results), using the domestic horse setting on both analyzers. These analyzers and settings have not been validated for elephant blood. Internal quality control using manufacturer-supplied quality control material was performed every day before analysis. These results from the original hematology analyses were reviewed for the present study. Firstly, the automated calculated hematocrit (HCT) was compared to a manual PCV performed at the same time. Only automated results with a HCT within 3% of the PCV were included in this study. The white blood cell count (WBC), red blood cell count (RBC) and platelet count (PLT) as measured by impedance (both analyzers), and the hemoglobin concentration (HGB), as measured by a cyanide-free photometric method (both analyzers) were considered accurate enough to be used for this study. The erythrocyte indices mean cell volume (MCV), mean cell hemoglobin (MCH) and mean cell hemoglobin concentration (MCHC) were calculated using the following standard equations:

$$\begin{array}{l} MCV=\frac{\left[PCV\;\times \;10\right]}{RBC};MCH=\frac{HGB}{RBC};MCHC=\frac{\left[HGB\;\times \;100\right]}{PCV} \end{array}$$

(Units: HGB g/L RBC × 10^12^/L, PCV %; MCV fL, MCH pg, MCHC g/L)

A 200-cell leukocyte differential count was performed on the available corresponding blood smears. Two smears could not be evaluated due to poor quality. Morphology of the erythron, leukon, and thrombon were recorded. The amount of rouleaux formation was graded from 0 to 4, where 0 was no rouleaux, 1 was the extension of rouleaux to 25% of the smear from the droplet end, 2 was extension to 50%, 3 was extension to 75% and 4 was extension of rouleaux all the way to the feathered edge. This scale was formulated by the authors to accommodate the high amount of rouleaux formation normally present in elephants. The number of heterophils with toxic changes was recorded (few, moderate, severe) as well as the severity of toxicity (1+ to 4+) according to a standardized grading system described for domestic species (23). Morphology of other leukocytes was recorded based on the descriptions for elephants found in Schalm's Veterinary Hematology (24) and the morphological changes reported by Stacy et al. (25). Monocytes were subclassified as regular monocytes, bilobed monocytes, and round monocytes, and each type was recorded separately. Monocytes were counted as bilobed when either two clearly separated nuclear lobes were visible or the isthmus connecting them was less than a third of the nucleus diameter. Characteristics used to define round monocytes were the almost complete roundness of their nuclei, a nucleus to cytoplasm ratio of 1:1, and coarser chromatin, compared to that of lymphocytes. Active lymphocytes were also recorded if there were more than 5% on a slide.

### Clinical Chemistry

Analysis was performed using the Abaxis VetScan VS2 (Abaxis, Union City, USA) according to the manufacturer's instructions. The Large Animal rotor (Abaxis, catalog number 500-0023-12, lot number 9214BC3) was used and included the following measurands: albumin, alkaline phosphatase (ALP), aspartate aminotransferase (AST), calcium, creatine kinase (CK), gamma glutamyltransferase (GGT), globulin, magnesium, phosphorus, total protein (TP) and urea. The analytical methods for these measurands are shown in Table 1. This profile was considered by veterinarians experienced in elephant medicine to be the most valuable for this species. Serum samples were thawed overnight at 4°C, then mixed using a vortex machine for 15–30 s, and thereafter pipetted into the sampling area of the rotor and analyzed. An automatic electronic quality control check is performed on each rotor before analysis. In order to obtain an estimate of analytical precision specifically for elephant serum, remaining serum was pooled, and this pool was measured twenty times (on twenty rotors from the same lot, number 9214BC3) in 1 day. The imprecision, represented by the coefficient of variation (CV), was calculated from the mean and standard deviation (SD) (CV% = SD/mean × 100).

**Table 1:** Assay methods utilized by the Abaxis VetScan VS2 for the analysis of African elephant serum samples.

**Measurand**	**Abaxis VetScan VS2**
Albumin	Bromocresol green dye-binding method
ALP	Kinetic (p-nitrophenol phosphate)
AST	Kinetic (l-aspartate and α-ketoglutarate)
Calcium	Arsenazo III method
CK	Kinetic (creatine phosphate)
GGT	Kinetic (L-γ-glutamyl-3-carboxy-4-nitroanilide)
Globulin	Calculated
Magnesium	Enzymatic (hexokinase)
Phosphate	Enzymatic (glucose-6-phosphate dehydrogenase)
Total protein	Biuret Method
Urea	Enzymatic (urease)

*ALP, alkaline phosphatase; AST, aspartate aminotransferase; CK, creatine kinase; GGT, gamma-glutamyltransferase*.

### Statistical Analysis

Statistical analyses were performed with MedCalc software version 19.1.7 (MedCalc Software, Ostend, Belgium) and the Excel add-on Reference Value (RefVal) Advisor version 2.1 (26) according to guidelines published by the ASVCP. Histograms were inspected visually, and Dixon and Tukey tests were used to identify outliers. If any measurand was identified as an outlier, the whole data set of this sample was excluded, and the statistical analysis rerun. This procedure was repeated until no more outliers remained, for any measurands. Normality was assessed using a Shapiro-Wilk test. Based on ASVCP guidelines for RI determination (7), the following strategies were then followed:

For all sample sizes, a cut-off *p*-value of >0.2 for the Shapiro-Wilk test, not >0.05, was used to define a Gaussian distribution (19). For samples sizes of 40–80, if *p* > 0.2, then the parametric method was used to generate the 95% reference limits. If *p* ≤ 0.2, the non-parametric method was used (7, 20). For sample sizes of 20–40 the RI were calculated with the parametric (*p* > 0.2) or robust method (*p* ≤ 0.2, data Box-Cox transformed). The 90% confidence intervals (CI) of the lower and upper reference limits (RL) were calculated using a bootstrap method. The ratio of the upper or lower CI to the RI was calculated by dividing the former by the latter (7).

To examine the potential effect of storage time on clinical chemistry measurands, the number of days from storage to analysis was enumerated, and the correlation between days in storage and measurand concentration or activity calculated using Pearson's correlation coefficient, *r* (*p* < 0.05 considered significant).

## Results

**Figure 1:**
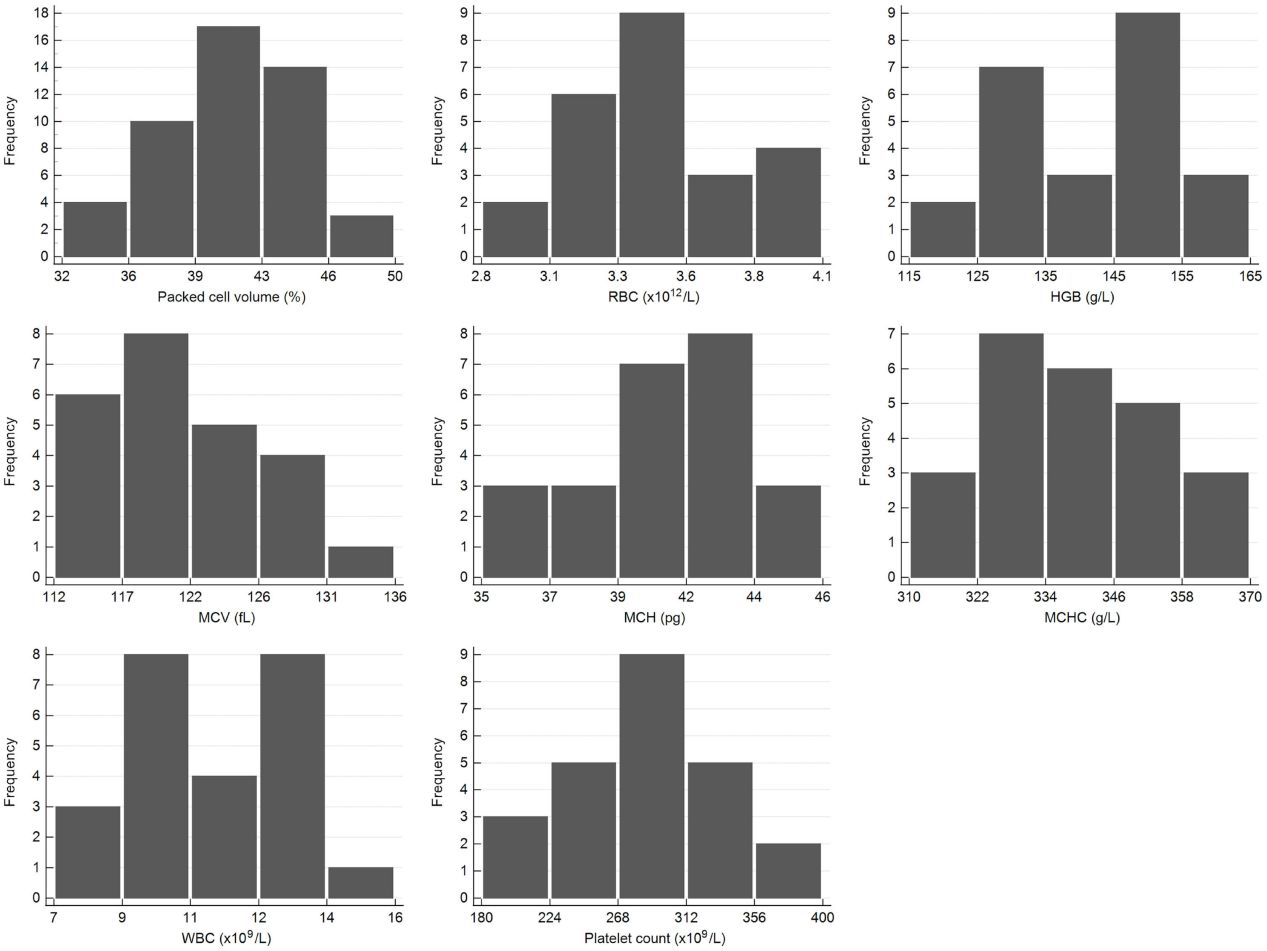
Histograms showing the distribution of results for selected hematology measurands from the African elephant (*Loxodonta africana*) for the scil Vet abc or Horiba ABX Micro ESV60. The x-axis represents the measurand result; the y-axis represents the frequency of these values occurring.

**Figure 2:**
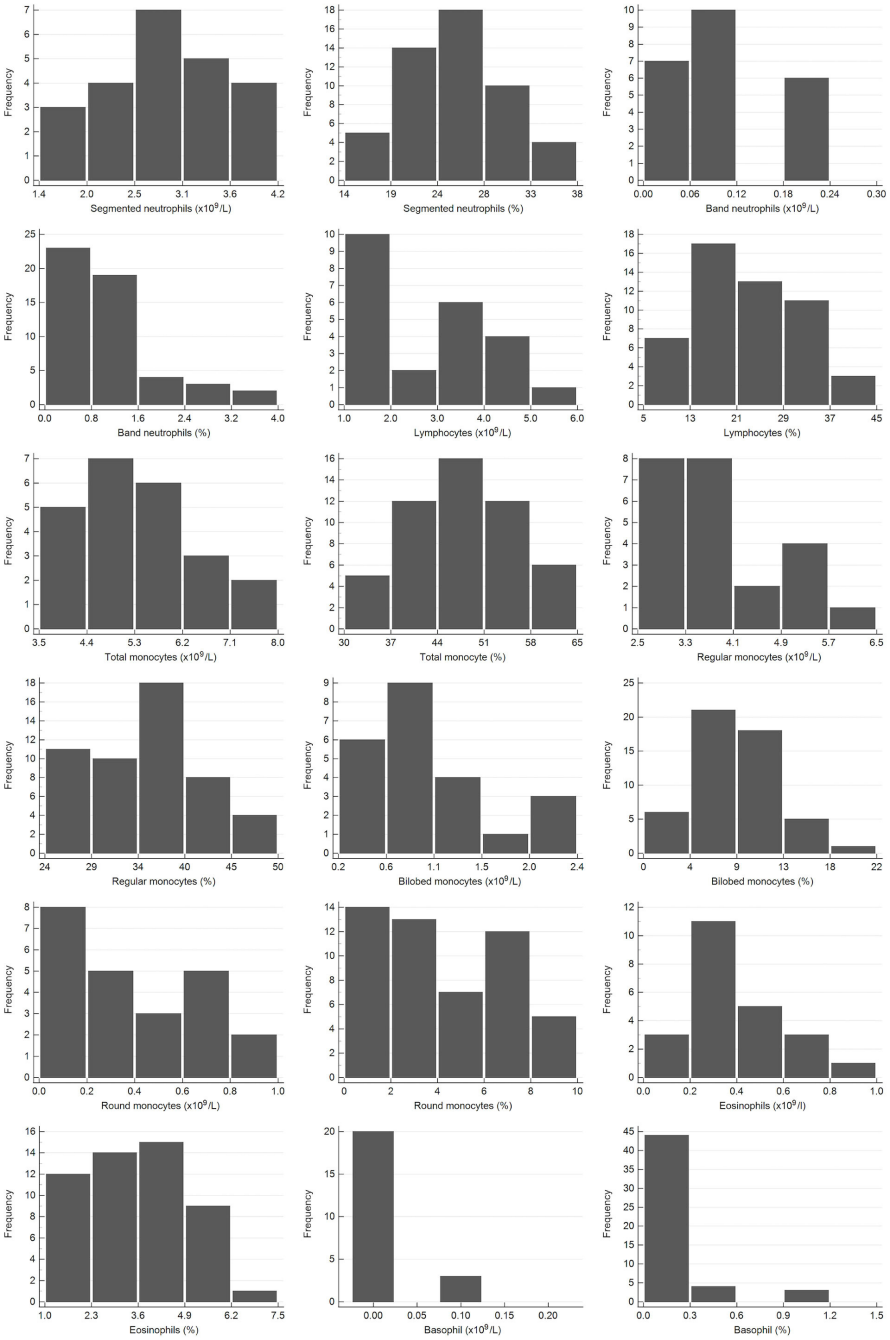
Histograms showing the distribution of results for leukocytes from the African elephant (*Loxodonta africana*) for the scil Vet abc or Horiba ABX Micro ESV60 and manual differential leukocyte counts. The x-axis represents the measurand result; the y-axis represents the frequency of these values occurring.

**Figure 3:**
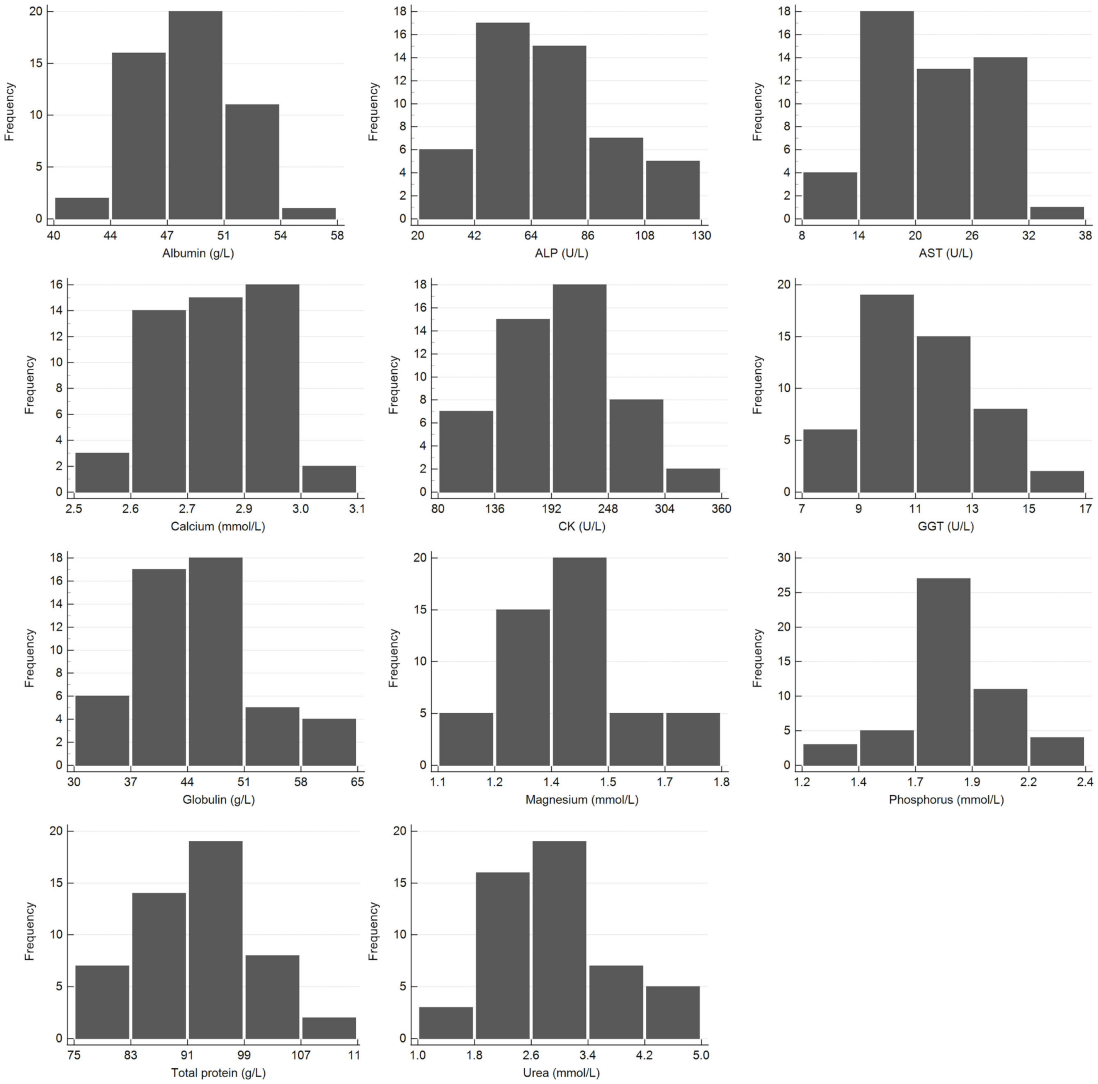
Histogram showing the distribution of results for clinical chemistry measurands from the African elephant (*Loxodonta africana*) for the Abaxis Vetscan VS2. The x-axis represents the measurand result; the y-axis represents the frequency of these occurring.

Samples were selected from free-ranging elephants, with no overt clinical abnormalities, living in the KNP in South Africa and were collected between October 2014 and August 2019. Of the original 79 selected samples, two were excluded after further detailed analysis of their capture records, as one was found deceased subsequent to a road traffic accident, and one had a snare injury. After statistical analysis and removal of 28 complete data sets with outliers, 51 samples from apparently healthy animals remained. These consisted of 42 males and 9 females. All were sub adults or adults according to the original selection datasheet, except for one male calf which was estimated to be 4 years old. Results for PCV were available for 48 of these animals. Only 23 samples from the automated hematology analysis were finally included, after applying the described screening procedures. Results thereof are shown in Table 2 and results for all manual relative differential leukocyte counts (n = 51) are presented in Table 3. Clinical chemistry RI were established from 50 individuals (one serum sample was missing) and are shown in Table 4. Mean, median, standard deviation as well as the distribution and chosen statistical method for all measurands are presented. Histograms for hematology and clinical chemistry are presented in Figures 1–3. Original data are presented in Supplementary Table 1.

**Table 2:** Hematology reference intervals from free-ranging African elephants (*Loxodonta africana*) of the Kruger National Park.

**Measurand (unit)**	***n***	**Mean**	**SD**	**Median**	**Min**	**Max**	**RI**	**LRL**	**URL**	**Distribution**	**Method**
PCV (%)	48	41	4	41	33	49	34–49	32–35	47–50	G	P
RBC (× 10^12^/L)	23	3.44	0.30	3.48	2.80	3.96	2.80–4.08	2.64–2.98^*^	3.90–4.26^*^	G	P
HGB (g/L)	23	141	13	143	116	163	114–167	107–121^*^	160–175^*^	G	P
MCV (fL)	23	121	6	121	112	134	109–133	106–113^*^	129–136^*^	G	P
MCH (pg)	23	40.9	2.6	41.4	35.5	45.2	35.5–46.3	34.2–37.0^*^	44.8–47.8^*^	G	P
MCHC (g/L)	23	338	15	337	314	364	306–370	298–315^*^	361–379^*^	G	P
PLT (× 10^9^/L)	23	284	54	281	182	386	171–398	142–203^*^	364–429^*^	G	P
WBC (× 10^9^/L)	23	11.2	2.2	11.3	7.5	15.2	6.6–15.7	5.4–7.8^*^	14.4–17.0^*^	G	P
Segmented heterophils (× 10^9^/L)	23	2.8	0.7	2.9	1.5	4.0	1.4–4.3	1.0–1.8^*^	3.8–4.7^*^	G	P
Band heterophils (× 10^9^/L)	23	0.1	0.1	0.1	0.0	0.2	0–0.6	0–0	0.4–0.8^*^	NG	Robust
Total monocytes (× 10^9^/L)	23	5.2	1.1	5.0	3.6	7.6	2.8–7.7	2.2–3.5^*^	6.9–8.3^*^	G	P
Monocytes normal (× 10^9^/L)	23	3.8	0.9	3.7	2.7	6.1	2.5–6.9	2.3–2.7	5.5–8.9^*^	NG	Robust
Monocytes bilobed (× 10^9^/L)	23	1.1	0.5	0.9	0.3	2.3	0.3–2.6	0.2–0.4	1.9–3.4^*^	NG	Robust
Monocytes round (× 10^9^/L)	23	0.3	0.3	0.3	0.0	0.9	0.0–1.7	0.0–0.0	1.3–2.3^*^	NG	Robust
Lymphocytes (× 10^9^/L)	23	2.7	1.3	2.3	1.1	5.5	0.7–7.0	0.5–1.1	5.4–9.2^*^	NG	Robust
Eosinophils (× 10^9^/L)	23	0.4	0.2	0.3	0.0	0.9	0.1–0.9	0.1–0.1	0.7–1.2^*^	NG	Robust
Basophils (× 10^9^/L)	23	0.0	0.0	0.0	0.0	0.1	0.0–0.1	Not able to calculate with any method			NP

* *The CI (confidence interval of the upper or lower reference limit) to RI ratio exceeded 20%*.

*PCV, packed cell volume; RBC, red blood cell concentration; HGB, hemoglobin concentration; MCV, mean cell volume; MCH, mean cell hemoglobin; MCHC, mean cell hemoglobin concentration; PLT, platelet concentration; WBC, white blood cell concentration; n, number of individuals; SD, standard deviation; RI, reference interval; LRL, 90% confidence interval of the lower reference limit; URL, 90% confidence interval of the upper reference limit; G, Gaussian; NG, non-Gaussian; P, parametric; NP, non-parametric*.

*PCV was measured by manual methods; WBC, RBC, HGB, and PLT were measured using a scil Vet abc or Horiba ABX Micro ESV60; MCV, MCH, and MCHC were calculated. Absolute leukocyte numbers were derived from WBC and 200-cell manual leukocyte differential counts*.

**Table 3:** Reference intervals for relative leukocyte differential counts for African elephants (*Loxodonta africana)* of the Kruger National Park.

**Leukocyte**	***n***	**Mean**	**SD**	**Median**	**Min**	**Max**	**RI**	**LRL**	**URL**	**Distribution**	**Method**
Segmented heterophils (%)	51	25.6	4.9	25.5	15.5	36.0	15.6–35.5	13.8–17.5	33.6–37.4	G	P
Band heterophils (%)	51	1.0	1.0	1.0	0.0	3.5	0.0–3.5	0.0–0.0	3.0–3.5	NG	NP
Total monocytes (%)	51	47.6	8.0	48.0	31.0	63.5	31.5–63.8	28.5–34.5	60.6–66.9	G	P
Monocytes normal (%)	51	35.2	6.5	35.0	24.0	49.0	22.1–48.3	19.7–24.5	45.7–50.8	G	P
Monocytes bilobed (%)	51	8.6	4.0	8.5	1.0	19.5	1.2–18.8	1.0–2.3	15.7–19.5^*^	G	NP
Monocytes round (%)	51	3.9	2.9	3.5	0.0	9.5	0.0–9.4	0.0–0.0	8.2–9.5	NG	NP
Lymphocytes (%)	51	22.2	8.9	23.0	6.0	40.0	7.4–39.9	6.0–10.7	36.5–40.0	NG	NP
Eosinophils (%)	51	3.5	1.4	3.5	1	7.0	1.2–6.7	1.0–1.7	5.7–7.0^*^	NG	NP
Basophils (%)	51	0.1	0.3	0.0	0.0	1.0	0.0–1.0	0.0–0.0	0.8–1.0	NG	NP

* *The CI (confidence interval of the upper or lower reference limit) to RI ratio exceeded 20%*.

*n, number of individuals; SD, standard deviation; RI, reference interval; LRL, 90% confidence interval of the lower reference limit; URL, 90% confidence interval of the upper reference limit; G, Gaussian; NG, non-Gaussian; P, parametric; NP, non-parametric*.

*200-cell manual differential counts were performed*.

**Table 4:** Serum clinical chemistry reference intervals from free-ranging African elephants (*Loxodonta africana*) of the Kruger National Park, using the Abaxis VetScan VS2.

**Measurand (unit)**	***n***	**Mean**	**SD**	**Median**	**Min**	**Max**	**RI**	**LRL**	**URL**	**Distribution**	**Method**
Albumin (g/L)	50	48	3	48	41	56	41–55	41–44^*^	52–56^*^	NG	NP
ALP (U/L)	50	68	23	64	28	124	30–122	28–38	115–124	NG	NP
AST (U/L)	50	21	6	21	9	36	9–34	6–11	31–36	G	P
Calcium (mmol/L)	50	2.79	0.11	2.78	2.55	3.04	2.56–3.02	2.52–2.61	2.98–3.07	G	P
CK (U/L)	50	203	58	200	86	341	85–322	63–107	298–345	G	P
GGT (U/L)	50	11	2	11	7	16	7–16	7–8	14–16^*^	NG	NP
Globulin (g/L)	50	45	7	44	31	60	30–59	28–33	56–62	G	P
Magnesium (mmol/L)	50	1.41	0.15	1.41	1.14	1.70	1.15–1.70	1.14–1.22	1.68–1.70	NG	NP
Phosphorus (mmol/L)	50	1.82	0.23	1.82	1.27	2.31	1.28–2.31	1.27–1.45	2.23–2.31	NG	NP
Total protein (g/L)	50	93	8	93	78	112	77–109	74–80	106–112	G	P
Urea (mmol/L)	50	2.9	0.8	3.0	1.0	4.8	1.2–4.6	0.9–1.	4.3–5.0	G	P

* *The CI (confidence interval of the upper or lower reference limit) to RI ratio exceeded 20%*.

*ALP, alkaline phosphatase; AST, aspartate aminotransferase; CK, creatine kinase; GGT, gamma-glutamyltransferase; n, number of individuals; SD, standard deviation; RI, reference interval; LRL, 90% confidence interval of the lower reference limit; URL, 90% confidence interval of the upper reference limit; G, Gaussian; NG, non-Gaussian; P, parametric; NP, non-parametric*.

The CVs obtained for the clinical chemistry measurands from the repeatability study with elephant serum on the VS2 were: albumin 1.1%, ALP 3.1%, AST 4.7%, calcium 0.8%, CK 5.2%, GGT 6.6%, globulin 3.7%, magnesium 0.9%, phosphorous 1.0%, TP 1.4%, urea 3.9%. Imprecision for all measurands was considered acceptable, when compared to total allowable analytical error guidelines for veterinary species (27).

No significant correlation between storage time (minimum 5 months to maximum 5 years) and measurand concentration/activity was found for any measurands apart from AST and CK (AST *r* = 0.69, *p* < 0.001; CK *r* = 0.50, *p* < 0.001). In other words, older samples appeared to have a decreased AST and CK activity compared to more recent samples, which may indicate a storage effect (28).

**Figure 4:**
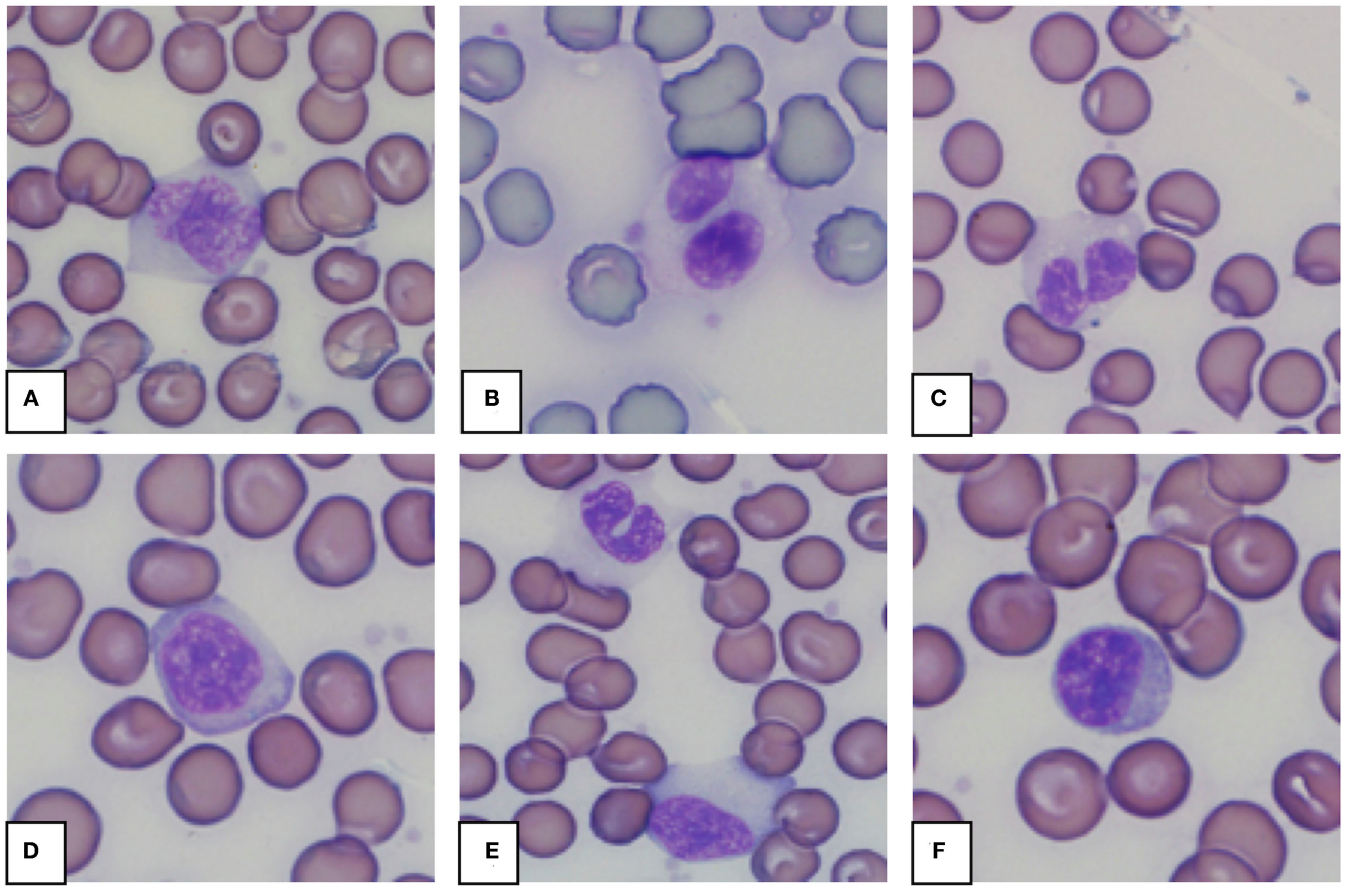
Representative monocytes **(A–E)** and lymphocyte **(F)** in blood smears from an African elephant (*Loxodonta africana*). **(A)**: Normal monocyte; **(B,C)**: Bilobed monocytes; **(D)**: Round monocyte; **(E)**: Normal and bilobed monocyte; **(F)**: Lymphocyte. Wright -Giemsa stain; 1,000x magnification.

**Figure 5:**
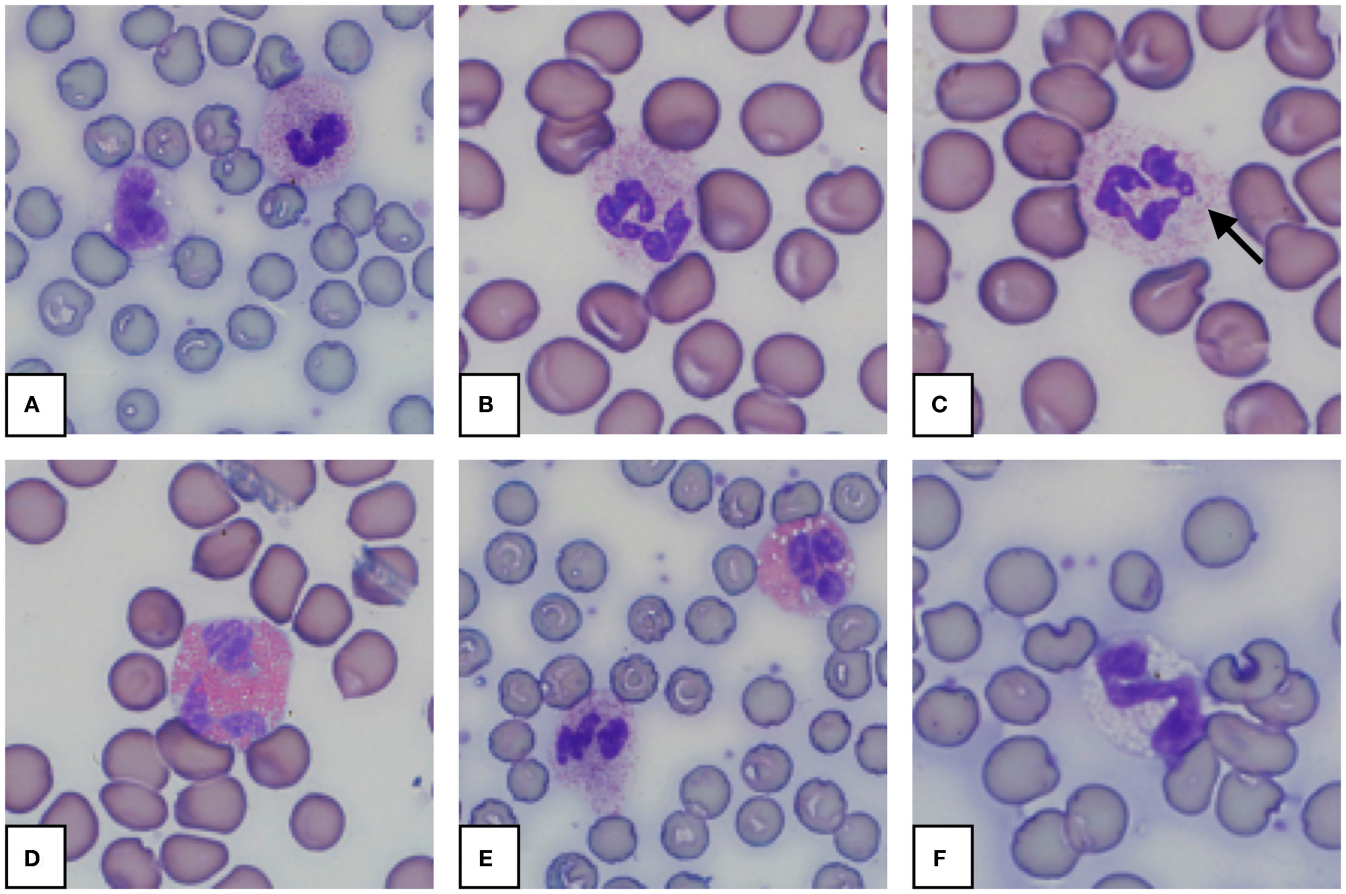
Representative granulocytes in blood smears from an African elephant (*Loxodonta africana*). **(A)**: Heterophil and normal monocyte; **(B)**: Heterophil; **(C)**: Heterophil with toxic changes (Dohle body, arrow); **(D)**: Eosinophil; **(E)**: Heterophil and eosinophil; **(F)**: Basophil. Wright -Giemsa stain; 1,000x magnification.

Morphology from the microscopic blood smear evaluation was recorded, with examples shown in Figures 4, 5. Rouleaux ranged from 2+ in 22 blood smears to 4+ in one blood smear. Mild anisocytosis was seen in over 90% of the evaluated smears. Band heterophils were seen in 31 of 51 blood smears. The number of heterophils showing toxic changes was categorized as few to moderate in 20 blood smears and high in 6 blood smears. In 16 of the 26 smears with toxic heterophils, dark blue-gray cytoplasm with vacuoles or toxic granulation was noted (3+) (Figure 5) and karyolysis was seen in one (4+ toxicity grade). Monocytes were divided into three categories: regular, bilobed and round (Figure 4). Normal monocytes were observed most commonly and round monocytes least commonly (Table 3 and Figure 4). Occasionally, trilobed monocytes were seen, which were counted as bilobed. Monocyte activity, characterized by the number of vacuoles, was mild in 41 smears and recorded as moderate in 6 smears. More than 5% of lymphocytes were reactive in 26 smears. Eosinophils (Figure 5) were characterized by bright red granules and were present in every slide. Basophils had a light blue to transparent cytoplasm with non-staining granules and were detected in only seven smears (Figure 5).

## Discussion

This study presents RI for hematology and clinical chemistry for the free-ranging African elephant, with the intent of providing valuable guideline data for clinicians working with the species. Hematology RIs were generated from automated analysis as well as from manual counts; the latter supplying useful data for situations where resources are limited. The selected clinical chemistry panel includes measurands that are applicable and useful for this species. For all RIs, a very strict outlier elimination approach was followed, as the samples originated from wild animals whose health status was presumed normal based on a cursory examination but could not be confirmed. It can be assumed that in the wild, a certain proportion of a population have an underlying subclinical disease, and strict outlier exclusion ensures that the resultant RIs are as representative of a truly healthy population as possible.

Reference interval studies for African elephants have been performed and described previously, albeit with various limitations for material and methods. While this is the second study for this specific geographic location and population, the previous study, published in 1984, generated data largely from animals that were shot prior to blood collection (29). Culled animals were similarly used in studies with larger sample sizes (*n* = up to 141), published between 1977 and 1980; all these data originated from the same elephant population in eastern Africa (12, 30–33). The same blood collection method was performed in a smaller Tanzanian study (*n* = 18–23), where on later examination, infestation with the bile duct hookworm (*Grammocephalus sp*.) and other parasites was discovered (11). Post mortem findings or coproscopic examinations were either not performed or reported for any of the other studies involving culled animals (12, 29, 34, 35). Two RI studies were performed on managed animals which were either chemically immobilized or trained to stand for blood collection (9, 10). In both, blood was collected *via* venipuncture from either the auricular or the saphenous vein and animals were reported to be clinically healthy. The limitations of the first of these studies are the analysis of a limited range of measurands, small sample size (*n* = 5) and a different subspecies of African elephant (*Loxodonta africana cyclotis*) (9). Allen et al. (10) report data from 31 animals for comparable measurands to our study, but all their elephants were young, between 4 and 8 years old, and the immobilization protocol varied amongst individuals. Most of the aforementioned studies used manual analytical methods for hematology, when methods were reported (9). Data were presented as mean or median with the standard deviation (SD) or range; outlier identification and exclusion were not performed and RI and CI of the RLs were not calculated.

Studies on Asian elephants are more common and involve both managed and free-ranging animals. Managed elephants often reside in tourist camps or are working elephants from the timber industry, from various locations in South East Asia, including India, Sri Lanka, Thailand and Myanmar (15, 17, 36). Blood was collected from the auricular vein without immobilization, except for one study on free-ranging elephants where a combination of etorphine and acepromazine was used (15). All blood samples were obtained from auricular veins with either 14G needles or vacutainer systems, but variation in sample handling, storage and transportation is described and analytical methods range from manual to completely automated. In most of these studies, presented data include hematology reference values, and at least some clinical chemistry measurands are presented, apart from one of the earliest studies, from elephants in India (36). Another study, using samples from zoo Asian elephants, focused on blood cell morphology using conventional light and electron microscopy and also describes cytochemical staining characteristics (37).

In the above mentioned studies on Asian elephants, the animals were described as clinically healthy or apparently healthy. Sample sizes varied greatly; the smallest one was performed on six individuals with longitudinal samples (38) with the largest sample size up to 765, although some of the animals were sampled two or three times (17). Silva et al. were able to compare domesticated with free-ranging animals, all originating from one geographical region and using the same study methods (15, 39). Most published results have been reported as means or medians, SDs and ranges. More recent studies include outlier detection and calculation of 90% CI (16, 17). Only the most recent publication, on Asian elephants, generated RI in accordance with the ASVCP guidelines, and performed partitioning for sexes and age groups (17). A few studies have also investigated the influence of musth on clinical chemistry measurands and hormone concentrations (16, 38).

Hematologic values have been shown to vary by geographical location. The means for RBC (3.9 × 10^12^) and PCV (49%) described in the East African study, were higher than those determined in the current study and the minimum-maximum (1.4–6.0 × 10^12^/L and 18–80%) ranges much wider, in comparison to the current reference intervals (33). Reasons for this could include the presence of underlying illnesses or dehydration, as no health assessment was performed prior to collection; as well as differing blood collection methods and preanalytical errors, the lack of outlier identification and subsequent exclusion of samples. Hematocrit values (39.3–47.7%) from KNP elephants, sampled from seven groups with different restraint methods (sedated to culled) were comparable to the current PCV findings, however, other hematology RIs were not given (29). Immobilized captive African elephants showed lower mean RBC (3.0 × 10^12^/L) and PCV (35.1%); MCV range (106–122 fL) was slightly narrower and MCHC range (310–390 g/L) wider but comparable to the current RIs (10). Asian elephants tend to have lower PCVs with lower hemoglobin concentrations and lower RBC counts than African elephants (15, 17, 39). However, the current established means of MCV (121 fL), MCHC (338 g/L) and MCH (41 pg) are almost the same as those reported in immobilized Asian elephants in Sri Lanka (15).

The WBC RIs generated in this study are found to be well within the middle of the ranges of data presented in other studies, where the lowest WBC count is 6.83 (× 10^9^/L), reported in the African forest elephant (*n* = 5) (9) and the highest is 13.6 (× 10^9^ /L) from captive and immobilized African elephants (10). Asian elephant WBC counts vary at least as widely amongst various studies and range from 8.8 (× 10^9^/L) in tuskers (36) to 18.0 (× 10^9^/L) in free-ranging elephants in Sri Lanka, the latter being the highest reported WBC count in apparently healthy elephants (15). The percentages described for leukocyte subpopulations vary greatly between studies, especially for monocytes and lymphocytes, due to discrepancies in the identification and classification of bilobed and round monocytes. The morphology of these monocytes is unique to the Paenungulata clade, which also includes the Procaviidae (hyraxes) and the Sirenia families (dugongs and manatees) (40, 41). Bilobed and round monocytes have been confirmed to be of monocytic origin based on cytochemical staining (10, 42). In the current study, the three types of monocytes were identified using morphological characteristics described in the more recent literature (25, 42). Presumably, bilobed as well as round monocytes have been counted as lymphocytes in multiple other studies, which resulted in lymphocyte percentages above 50%, up to 77% (11, 33, 36). Two studies involving captive and free-ranging Asian elephants identified the bilobed monocyte as such, but the reported lymphocyte counts still appeared to be high, with means of 38% for managed, and 44% for free-ranging elephants (15, 39). Exclusion of samples and handling of outliers were not described. Possible reasons for the high proportion of lymphocytes are that round monocytes were counted as lymphocytes, and that animals with subclinical illnesses were included.

Elephants share another hematological characteristic with sirenians, in that these species have heterophils rather than neutrophils (41). Band heterophils have been described in low numbers in Asian elephants (15, 37, 39), but there is disagreement as to whether they occur in healthy animals (42). They have only been described in one study on African elephants (1.17%), but these animals were known to have parasites (11). The presence of band heterophils, and heterophils with toxic change, in over half of the elephants in this study indicates that low-grade inflammatory disease, not apparent during clinical examination, was possibly present in many of the current reference individuals. The current findings indicate that band heterophils of up to 3.5% can be expected for apparently healthy KNP elephants. The potential causes of this tendency toward a left shift need to be investigated further.

Eosinophils and basophils have been reported by most authors in low and comparable numbers to those here; except that eosinophil concentrations from managed African elephants (mean of 0.05 × 10^9^/L) are lower than the current RIs (0.4 × 10^9^/L) (10). This could reflect a higher parasite load in the free-ranging, compared to the managed population. Similar percentages of eosinophils and basophils were described for free-ranging and domesticated Asian elephants with 5% for eosinophils and 0.03% for basophils (15, 39). Interestingly, the most recent study on elephants in Myanmar does not report findings on either basophils or thrombocytes (17).

Platelets are the cell fraction with the widest ranges, as described in most studies where platelets were assessed (15, 16, 38, 39). They occur in large numbers but are very small in size and often found as clumped aggregates, making it difficult to identify single cells. The current range determined for the KNP elephant population is narrower and lower than for other African elephants (294–455 × 10^9^/L) (42). The lowest mean number for platelets was found for domestic Asian elephants in Sri Lanka (215 × 10^9^/L) (39) which remains within the range established for elephants in Thailand (101–590 × 10^9^/L) (16). The highest reported count is 719 × 10^9^/L, also found in the Asian elephant (42). The wider ranges reported in other studies may be due to the presence of platelet clumping, differing analytical methods and the lack of outlier exclusion.

Most of the results for clinical chemistry measurands reported in other studies of comparable geographical region were similar to the current measurements, even though analytical methods differed (12, 29, 32, 43). Reported means and medians were mostly within the current RIs. Seasonal changes could be seen for some analytes and other authors reported variations between age groups especially for minerals (32, 34). Differences can be seen in data published from managed African elephants, although sample size and age (4–8 years) need to be considered when making direct comparisons (10). Differences between sexes seem more prominent in the Asian elephant than in the African elephant, especially for serum activities of ALP and GGT which are much higher during musth periods (17, 38). The majority of measurands included in our panel have also been studied in the Asian elephant: total protein or protein fractions tend to be included in different studies as well as selected enzymes, while magnesium and GGT have not been widely investigated (16, 17, 39). Veterinarians with clinical experience of these species advise the inclusion of fibrinogen, creatinine, lactate dehydrogenase and electrolytes (Na, K, Cl) in clinical chemistry panels for elephants, in addition to the current study measurands (44). However, these were not included in our study due to the set profile of the VetScan VS2 rotor system.

Serum mineral concentrations in the African elephant vary by geographical location. The means of calcium, magnesium and phosphorus in the current study were higher compared to those from elephants at Sengwa Wildlife Research, Zimbabwe, but lower in comparison to elephants from Ruwenzori National Park in Uganda (29, 30). Firstly, a contributing factor that may explain the higher mineral concentrations and at least phosphorus in the Ruwenzori study could be hemolysis, as these elephants were bled after being culled. Secondly, nutrition may play a role; it has been shown previously that season and location have an influence on mineral and protein concentrations in blood (30, 32, 43). The blood samples in the current study were collected almost all year round, except during the months of January and February. One study showed that calcium and phosphorus were higher in male elephants and phosphorus was higher in young animals compared to animals above 5 years (34). In Asian elephants in Myanmar, calcium was lower (2.15–2.75 mmol/L), means of phosphorus and magnesium of managed Asian elephants were also lower than the current established means (17, 36). Blood mineral concentration data collected for zoo-housed African elephants also showed lower means than the KNP population (45). Seasonal and geographical, and therefore nutritional, variation will need to be taken into consideration when using the reference intervals in the future, as elephants tend to graze more during the wet season, when grassland is lush. In zoos, this information could be used as an aid for evaluating nutritional status and preventing mineral deficiencies which could influence bone health.

Determination of AST and CK activity is especially important for captured animals, as increases in these enzymes are associated with muscle injury, intramuscular injection, trauma and capture stress in domestic and wild animals such as dogs, horses, some ruminants and rhinoceros (46–48). Animals known to be injured were excluded from the current study. Kock et al. described a difference in the activities of AST and CK depending on etorphine dosages and the time to recumbency that elephants experienced during capture (43). The capture protocol with the higher etorphine dosages led to quicker recumbencies and lower AST and CK, compared to a lower dose protocol with longer times to recumbency. Although with both protocols, measurements were within the current reference intervals, AST and CK for the higher dosed group were visibly lower; duration of chasing and capture must be considered as both enzymes rise within hours of the insult. CK reaches its peak after 24–36 h and declines thereafter. As blood samples from the current study elephants were drawn immediately after the animals were recumbent, results reflect CK activity well before its peak. Distinct increases in activities of both enzymes are usually seen after long transports or exhaustion, exercise and rhabdomyolysis (46). Reference intervals generated for AST from managed African elephants are very similar to the current ones, while other enzymes (ALP, GGT, and CK) show much broader RIs (45). Young animals, also true for elephants, have higher ALP levels, due to increased osteoblast activity. In male Asian elephants, ALP is especially elevated during musth periods (30, 38). This would need to be evaluated in future studies to determine if this also occurs in African elephants. Unfortunately, the presence of musth in the male elephants in our population was not recorded.

Published means of TP and albumin are consistently lower than those found in our study, for wild African elephants from various geographical locations (32, 43). Results from managed *Loxodonta africana* are even lower whereas urea and globulin means were higher than the current results (10, 45). Total protein reported for the older study of elephants from KNP was most similar to the current findings (29). Seasonal and geographical changes have been recorded for all protein constituents, pointing to nutrition as the most important variable (32). This needs to be considered when comparing results to established RIs. Total protein values from Asian elephant populations were similar to each other (65–93 g/L), and generally lower than TP in African elephants (15–17, 39).

Anesthetic drugs, stress, capture and transport or a combination thereof can lead to PCV and TP changes in various species, and thus influence RIs (49–51). In other animal species, stress during capture can lead to an increase in PCV and HGB via catecholamine release and contraction of the spleen (52). In the adult African elephant, the spleen acts mostly as a filtering organ (rather than as a blood storage organ) as is the case in animals which flee if they encounter enemies or hazards, like equids with a splenic capsule with muscle tissue (44). The elephant's splenic capsule consists of connective tissue, therefore a contraction as stress response seems unlikely (44). The anesthetic drugs etorphine and azaperone or combinations thereof have been commonly used for the African elephant as well as other wildlife species (53–56). Cardiopulmonary measurands, such as heart and respiratory rate, blood pressure, blood gases and hemoglobin in arterial blood have been described for the African elephant anesthetized with these drugs (57). The influence of these agents on clinical pathology has not been evaluated in this species so far. In one small antelope study, where etorphine was one component of the immobilization drugs (but not used on its own or with azaperone), a decrease in RBC, WBC, PCV, and HGB was found (58). A RI study on African buffalo (*Syncerus caffer*) from the same geographical location (KNP), using the same drug combination and clinical chemistry panel, did not consider the influence of the anesthetics relevant (59). One reason for this could be that blood is collected very soon after recumbency which does not allow enough time for measurands to react. This is a general assumption which needs to be studied further, for example by comparing results from samples taken under similar conditions and analyzed using the same methods, from non-immobilized and immobilized captive elephants. As most managed elephants are not immobilized for blood collection, the possible influence of drugs on the RIs generated in this study needs to be considered if these RIs are used to evaluate results from captive individuals.

These RIs were generated from a free-ranging elephant population, meaning that most likely not all reference individuals were healthy, as indicated by the presence of band heterophils and some toxic changes. It is difficult to determine health under field conditions as only certain parameters are assessable and the animals are chemically restrained. Generally, wild animals show signs of illness very late in the progression of a disease. Parasitic loads will likely also differ from managed animals, as fecal examinations and prophylactic deworming schedules are commonly recommended practices (60). The above described strict outlier elimination was performed to exclude most potential “unhealthy” animals and gain the cleanest possible data set. Reference intervals were established from the KNP population, and this should be taken into account when applying them to populations from a different geographical distribution or to managed individuals. However, these RIs could be particularly useful in terms of assessing the nutritional management of zoo or sanctuary animals as they are derived from a population existing in the natural habitat of this species, with free choice of fodder.

Another limitation of the current study could be the age of the bio-banked samples, which were stored up to 8 years at −80°C. The literature suggests that human serum samples can be stored for at least 13 months and plasma for up to 5 years at the same temperature (−80°C) without significant changes in the measurands (61, 62). In the current study, we found that the activity of the two enzymes, AST and CK, were lower in the older samples, which could be an indication of a storage effect (28). This would have influenced the RIs by resulting in falsely lower reference limits. However, as low activity of these enzymes is not considered clinically relevant, this limitation is not likely to have an effect on the clinical utility of the RIs.

For future studies, the sample size should be increased, especially for the automated hematology analyses, as the current sample size was rather small and the analyzers are not validated for this species. Ideally, the same machine should be used for all samples, which in the best case scenario is validated for the species to be analyzed. Unfortunately, this was not possible for this study due to resource restrictions present in the laboratory. It is important to note that the automated hematology reference intervals are specific to the analyzers and settings used in this study. The desirable sample size for all measurands would be >120, but above 39 is considered reasonable for RI studies, which we were able to attain for blood smear analyses and WBC count as well as chemistry measurands (7). Larger sample sizes would allow for partitioning of sexes, which was not possible here. Male samples are overly represented here, as most samples were obtained opportunistically during the course of other studies, which may have had selection criteria favoring bull elephants.

## Conclusion

This is the first RI study for hematology and a clinical chemistry panel relevant for the African elephant performed using appropriate statistical methods and a strict outlier elimination approach. In comparison with previously performed research on the same species and for Asian elephants, the current ranges are narrower, which will have a greater potential to identify normal and abnormal clinical pathology results in individuals of this species in the future. The established RI will function as an important tool for researchers and clinicians working with the species, provided that drugs, geographic location and nutrition are taken into consideration when interpreting results. Future studies will be needed with greater sample sizes to create RIs for different groups (male vs. female, adult vs. juvenile).

## Data Availability Statement

The original contributions presented in the study are included in the article/Supplementary Material, further inquiries can be directed to the corresponding author/s.

## Ethics Statement

The animal study was reviewed and approved by Veterinary Science Research Ethics Committee and Animal Ethics Committee (certificate number REC 132-19), Faculty of Veterinary Science, University of Pretoria.

## Author Contributions

CS contributed to conceptualization and study design, data collection, data curation, performed the data analysis, and wrote the first draft of the manuscript. EH contributed to conceptualization and study design, data collection, data curation, performed the data analysis, and acquired funding. JH contributed to data collection. MM contributed to conceptualization and design of the study, and assisted with sample provision. PB assisted with sample provision and data curation. All authors contributed to manuscript revision, and read and approved the submitted version.

## Conflict of Interest

The authors declare that the research was conducted in the absence of any commercial or financial relationships that could be construed as a potential conflict of interest.

## References

[1] Blanc JJ. Loxodonta africana. The IUCN Red List of Threatened Species (2008) e.T12392A3339343. 10.2305/IUCN.UK.2008.RLTS.T12392A3339343.en

[2] Thouless CR Dublin HT Blanc JJ Skinner DP Daniel TE Taylor RD. African Elephant Status Report 2016: An Update From the African Elephant Database. Occasional Paper Series of the IUCN Species Survival Commission, No. 60 IUCN / SSC Africa Elephant Specialist Group. Gland, Switzerland IUCN (2016) vi + 309p. Available online at: https://www.iucn.org/content/african-elephant-status-report-2016-update-african-elephant-database (accessed July 22, 2020).

[3] Graf RJP Fritsche M Schmidt J Mantei R Peter S Spangenberg F. Afrikanischer Steppenelefant (Loxodonta africana). Available online at: www.zootierliste.de (accessed March 24, 2019).

[4] Modise A. Minister Nomvula Mokonyane Highlights Progress on the Implementation of the Integrated Strategic Management of Rhinoceros and Other Associated Endangered Species in SA. (2019). Available online at: https://www.environment.gov.za/mediarelease/mokonyane_2018integratedstrategic_managementofrhinoceros_2019feb (accessed February 13, 2019).

[5] Koehl D. The Elephant Database; Elephant Data and Information Since 1995. (1995). Available online at: www.elephant.se (accessed March 29, 2019).

[6] Sukumar R. A brief review of the status, distribution and biology of wild Asian elephants *Elephas maximus*. Int Zoo Yearb. (2006) 40:1–8. 10.1111/j.1748-1090.2006.00001.x

[7] Friedrichs KR Harr KE Freeman KP Szladovits B Walton RM Barnhart KF. ASVCP reference interval guidelines: determination of *de novo* reference intervals in veterinary species and other related topics. Vet Clin Pathol. (2012) 41:441–53. 10.1111/vcp.12006 23240820

[8] Geffre A Friedrichs K Harr K Concordet D Trumel C Braun JP. Reference values: a review. Vet Clin Pathol. (2009) 38:288–98. 10.1111/j.1939-165X.2009.00179.x 19737162

[9] Woodford MH. Blood characteristics of the African elephant (*Loxodonta africana cyclotis*). J Wildl Dis. (1979) 15:111–3. 10.7589/0090-3558-15.1.111 459036

[10] Allen JL Jacobson ER Harvey JW Boyce W. Hematologic and serum chemical values for young African elephants (*Loxodonta africana*) with variations for sex and age. J Zoo Anim Med. (1985) 16:98–101. 10.2307/20094754

[11] Debbie JG Clausen B. Some hematological values of free-ranging African elephants. J Wildl Dis. (1975) 11:79–82. 10.7589/0090-3558-11.1.79 1113444

[12] Brown IRF White PT. Elephant blood haematology and chemistry. Comp Biochem Phys B. (1980) 65:1–12. 10.1016/0305-0491(80)90107-8

[13] Miller M Chen TC Holick MF Mikota S Dierenfeld E. Serum concentrations of calcium, phosphorus, and 25-hydroxyvitamin D in captive African elephants (*Loxodonta africana*). J Zoo Wildl Med. (2009) 40:302–5. 10.1638/2008-0098.1 19569477

[14] van Sonsbeek GR van der Kolk JH van Leeuwen JP Everts H Marais J Schaftenaar W. Effect of calcium and cholecalciferol supplementation on several parameters of calcium status in plasma and urine of captive Asian (*Elephas maximus*) and African elephants (*Loxodonta africana*). J Zoo Wildl Med. (2013) 44:529–40. 10.1638/2010-0123R4.1 24063079

[15] Silva ID Kuruwita VY. Hematology, plasma, and serum biochemistry values in free-ranging elephants (*Elephas maximus ceylonicus*) in Sri Lanka. J Zoo Wildl Med. (1993) 24:434–9.

[16] Janyamethakul TSS Somgird C Pongsopawijit P Panyapornwithaya P Klinhom S Loythong J. Hematologic and biochemical reference intervals for captive Asian elephants (*Elephas maximus*) in Thailand. Kafkas Univ Vet Fak. (2017) 23:665–8. 10.9775/kvfd.2017.17380

[17] Santos DJFD Jackson J Phil M Aung HH Nyein UK Htut W. Sex differences in the reference intervals of health parameters in semicaptive Asian elephants (*Elephas maximus*) from Myanmar. J Zoo Wildl Med. (2020) 51:25–38 10.1638/2018-0181 32212543

[18] Teare JA. ISIS, medARKS, ZIMS, and global sharing of medical information by zoological institutions. In: Miller REFM, editor. Fowler's Zoo and Wild Animal Medicine. Vol. 8. St. Louis: Elsevier Saunders Inc. (2014). p. 276–9.

[19] Le Boedec K. Sensitivity and specificity of normality tests and consequences on reference interval accuracy at small sample size: a computer-simulation study. Vet Clin Pathol. (2016) 45:648–56. 10.1111/vcp.12390 27556235

[20] Le Boedec K. Reference interval estimation of small sample sizes: a methodologic comparison using a computer-simulation study. Vet Clin Pathol. (2019) 48:335–46. 10.1111/vcp.12725 31228287

[21] Weiser G. Chapter 1 – General principles of laboratory testing and diagnosis. In: Thrall MA Weiser G Allison RW Campbell TW editors. Veterinary Hematology and Clinical Chemistry. Ames: Wiley-Blackwell (2012).

[22] Cray C Rodriguez M Zaias J Altman NH. Effects of storage temperature and time on clinical biochemical parameters from rat serum. J Am Assoc Lab Anim Sci. (2009) 48:202–4. 19383219 PMC2679668

[23] Harvey JW. Chapter 1 - Examination of blood samples. In: Harvey JW editor. Atlas of Veterinary Hematology. Philadelphia: W.B. Saunders (2001). p. 3–20.

[24] Weiss DJ Wardrop KJ. Schalm's Veterinary Hematology. Ames: Wiley-Blackwell (2010).

[25] Stacy NI Isaza R Wiedner E. First report of changes in leukocyte morphology in response to inflammatory conditions in Asian and African elephants (*Elephas maximus* and *Loxodonta africana*). PLoS ONE. (2017) 12:e0185277. 10.1371/journal.pone.0185277 28934325 PMC5608365

[26] Geffre A Concordet D Braun J-P Trumel C. Reference value advisor: a new freeware set of macroinstructions to calculate reference intervals with Microsoft Excel. Vet Clin Pathol. (2011) 40:107–12. 10.1111/j.1939-165X.2011.00287.x 21366659

[27] Harr KE Flatland B Nabity M Freeman KP. ASVCP guidelines: allowable total error guidelines for biochemistry. Vet Clin Pathol. (2013) 42:424–36. 10.1111/vcp.12101 24320779

[28] Gisslefoss RE Grimsrudd TK Morkrid L. Stability of selected serum proteins after long term storage in the Janus Serum bank. Clin Chem Lab Med. (2009) 47:596–603. 10.1515/CCLM.2009.121 19290843

[29] Hattingh J Wright PG de Vos V McNairn IS Ganhao MF Silove M. Blood composition in culled elephants and buffaloes. J S Afr Vet Assoc. (1984) 55:157–64. 6533304

[30] Brown IRF White PT. Serum calcium, magnesium, phosphorus and alkaline phosphatase in the African elephant, *Loxodonta africana*. Comp Biochem Phys B. (1977) 56:159–62. 10.1016/0305-0491(77)90042-6 830482

[31] Brown IRF White PT. Serum electrolytes, lipids and cortisol in the African elephant, *Loxodonta africana*. Comp Biochem Phys A. (1979) 62:899–901. 10.1016/0300-9629(79)90025-2

[32] Brown IRF White PT Malpas RC. Proteins and other nitrogenous constituents in the blood serum of the African elephant, *Loxodonta a fricana*. Comp Biochem Phys A. (1978) 59:267–70. 10.1016/0300-9629(78)90159-7

[33] White PT Brown IRF. Haematological studies on wild African elephants (*Loxodonta africana*). J Zool. (1978) 185:491–503. 10.1111/j.1469-7998.1978.tb03347.x

[34] Hill F Smith D. Clinical chemistry values for free-ranging elephants (*Loxodonta africana*) in Hwange National Park, Zimbabwe. Zimb Vet J. (1990) 21:33–42.

[35] Brown IRF White PT. Serum enzyme activities in the African elephant (*Loxodonta africana*). Experientia. (1976) 32:980–2. 10.1007/BF01933923 955033

[36] Nirmalan G Nair SG Simon KJ. Hematology of the Indian elephant (*Elephas maximus*). Can J Physiol Pharm. (1967) 45:985–91 10.1139/y67-116 6064061

[37] Salakij J Salakij C Narkkong N-A Apibal S Suthunmapinuntra P Rattanakukuprakarn J. Hematology, cytochemistry and ultrastructure of blood cells from Asian elephant (*Elephas maximus*). Witthayasan Kasetsat. (2005) 39:482–93.

[38] Niemuller C Gentry PA Liptrap RM. Longitudinal study of haematological and biochemical constituents in blood of the Asian elephant (*Elephas maximus*). Comp Biochem Phys A. (1990) 96:131–4. 10.1016/0300-9629(90)90053-U 1975529

[39] Silva ID Kuruwita VY. Hematology, plasma, and serum biochemistry values in domesticated elephants (*Elephas maximus ceylonicus*) in Sri Lanka. J Zoo Wildlife Med. (1993) 24:440–4.

[40] Aroch I King R Baneth G. Hematology and serum biochemistry values of trapped, healthy, free-ranging rock hyraxes (*Procavia capensis*) and their association with age, sex, and gestational status. Vet Clin Pathol. (2007) 36:40–8. 10.1111/j.1939-165X.2007.tb00180.x 17311193

[41] Harvey JW Harr KE Murphy D Walsh MT Nolan EC Bonde RK. Hematology of healthy Florida manatees (*Trichechus manatus*). Vet Clin Pathol. (2009) 38:183–93. 10.1111/j.1939-165X.2009.00113.x 19490571

[42] Harr KE Isaza R Blue JT. Hematology of elephants. In: Douglas J Weiss KJW editors. Schalm's Veterinary Hematology. 6th ed. Ames, Iowa: Blackwell Publishing Ltd. (2010). p. 942–9.

[43] Kock MD Martin RB Kock N. Chemical immobilization of free-ranging African elephants (*Loxodonta africana*) in Zimbabwe, using etorphine (M99) mixed with hyaluronidase, and evaluation of biological data collected soon after immobilization. J Zoo Wildl Med. (1993) 24:1–10.

[44] Mikota S. Hemolymphatic System. In: Fowler ME Mikota S editors. Biology, Medicine, and Surgery of Elephants. Ames, Iowa: Blackwell Publishing (2006). p. 325–45.

[45] Zims Expected Test Results for Loxodonta africana. Species360 Zoological Information Management System. Available online at: http://zims.Species360.org filters: No_selection_by_gender_All_ages_combined_Standard_International_Units_2013_CD (accessed September 2, 2017).

[46] Kaneko J Harvey J Bruss M. Clinical Biochemistry of Domestic Animals. 6th ed. San Diego, CA: Academy Press (2008). 138 p.

[47] Marco I Vinas L Velarde R Pastor J Lavin S. Effects of capture and transport on blood parameters in free-ranging mouflon (*Ovis ammon*). J Zoo Wildl Med. (1997) 28:428–33. 9580218

[48] Kock MD du Toit R Kock N Morton D Foggin C Paul B. Effects of capture and translocation on biological parameters in free-ranging black rhinoceroses (*Diceros bicornis*) in Zimbabwe. J Zoo Wildl Med. (1990) 21:414–24.

[49] Szabo MP Matushima ER de Castro MB Santana DA de Paula CD Duarte JM. Hematology of free-living marsh deer (*Blastocerus dichotomus*) from southeast Brazil. J Zoo Wildl Med. (2005) 36:463–9. 10.1638/04-404.1 17312766

[50] Presidente PJA Lumsden JH Presnell KR Rapley WA McCraw BM. Combination of etorphine and xylazine in captive white-tailed deer: II. Effects on hematologic, serum biochemical and blood gas values. J Wildl Dis. (1973) 9:342–8. 10.7589/0090-3558-9.4.342 4784323

[51] Knowles TG Warriss PD Brown SN Edwards JE Watkins PE Phillips AJ. Effects on calves less than one month old of feeding or not feeding them during road transport of up to 24 hours. Vet Rec. (1997) 140:116–24. 10.1136/vr.140.5.116 9042695

[52] Marco I Lavin S. Effect of the method of capture on the haematology and blood chemistry of red deer (*Cervus elaphus*). Res Vet Sci. (1999) 66:81–4. 10.1053/rvsc.1998.0248 10208884

[53] Still J Raath JP Matzner L. Respiratory and circulatory parameters of African elephants (*Loxodonta africana*) anaesthetised with etorphine and azaperone. J S Afr Vet Assoc. (1996) 67:123–7. 9120854

[54] Gaudio E Laubscher LL Pfitzer S Raath JP Hoffman LC De Benedictis GM. Immobilisation quality and cardiopulmonary effects of etorphine alone compared with etorphine-azaperone in blesbok (*Damaliscus pygargus phillipsi*). Vet Anaesth Analg. (2020) 47:528–36 10.1016/j.vaa.2019.10.012 32507718

[55] Vitali F Kariuki EK Mijele D Kaitho T Faustini M Preziosi R. Etorphine-azaperone immobilisation for translocation of free-ranging Masai giraffes (*Giraffa camelopardalis tippelskirchi*): a pilot study. Animals. (2020) 10:322. 10.3390/ani10020322 32085568 PMC7070639

[56] Buss P Miller M Fuller A Haw A Wanty R Olea-Popelka F. Cardiovascular effects of etorphine, azaperone, and butorphanol combinations in chemically immobilized captive white rhinoceros (*Cerathotherium simum*). J Zoo Wildl Med. (2016) 47:834–43. 10.1638/2015-0298.1 27691950

[57] Schumacher J Heard DJ Caligiuri R Norton T Jacobson ER. Comparative effects of etorphine and carfentanil on cardiopulmonary parameters in juvenile African elephants (*Loxodonta africana*). J Zoo Wildl Med. (1995) 26:503–7.

[58] Drevemo S Karstad L. The effect of xylazine and xylazine-etorphine-acepromazine combination on some clinical and haematological parameters in impala and eland. J Wildl Dis. (1974) 10:377–83. 10.7589/0090-3558-10.4.377 4436924

[59] Couch CE Movius MA Jolles AE Gorman ME Rigas JD Beechler BR. Serum biochemistry panels in African buffalo: defining reference intervals and assessing variability across season, age and sex. PLoS ONE. (2017) 12:e0176830. 10.1371/journal.pone.0176830 28472180 PMC5417560

[60] Mikota S. Preventive health care and physical examination. In: Fowler ME Mikota SK editors. Biology, Medicine and Surgery of Elephants. Ames, IA: Blackwell Publishing (2006). p. 67–73.

[61] Brinc D Chan MK Venner AA Pasic MD Colantonio D Kyriakopolou L. Long-term stability of biochemical markers in pediatric serum specimens stored at−80 °C: a CALIPER Substudy. Clin Biochem. (2012) 45:816–26. 10.1016/j.clinbiochem.2012.03.029 22510430

[62] Clark S Youngman LD Palmer A Parish S Peto R Collins R. Stability of plasma analytes after delayed separation of whole blood: implications for epidemiological studies. Int J Epidemiol. (2003) 32:125–30. 10.1093/ije/dyg023 12690023

